# Molecular characterization of a fungal gene paralogue of the penicillin *penDE *gene of *Penicillium chrysogenum*

**DOI:** 10.1186/1471-2180-9-104

**Published:** 2009-05-26

**Authors:** Carlos García-Estrada, Inmaculada Vaca, Ricardo V Ullán, Marco A van den Berg, Roel AL Bovenberg, Juan Francisco Martín

**Affiliations:** 1 Instituto de Biotecnología (INBIOTEC), Parque Científico de León, Av. Real, 1, 24006, León, Spain; 2 DSM Anti-Infectives, DSM Gist (624-0270), PO Box 425, 2600 AK, Delft, the Netherlands; 3 Área de Microbiología, Departamento de Biología Molecular, Facultad de CC Biológicas y Ambientales, Universidad de León, Campus de Vegazana s/n. 24071, León, Spain

## Abstract

**Background:**

*Penicillium chrysogenum *converts isopenicillin N (IPN) into hydrophobic penicillins by means of the peroxisomal IPN acyltransferase (IAT), which is encoded by the *penDE *gene. *In silico *analysis of the *P. chrysogenum *genome revealed the presence of a gene, Pc13g09140, initially described as paralogue of the IAT-encoding *penDE *gene. We have termed this gene *ial *because it encodes a protein with high similarity to IAT (IAL for IAT-Like). We have conducted an investigation to characterize the *ial *gene and to determine the role of the IAL protein in the penicillin biosynthetic pathway.

**Results:**

The IAL contains motifs characteristic of the IAT such as the processing site, but lacks the peroxisomal targeting sequence ARL. Null *ial *mutants and overexpressing strains indicated that IAL lacks acyltransferase (penicillin biosynthetic) and amidohydrolase (6-APA forming) activities *in vivo*. When the canonical ARL motif (leading to peroxisomal targeting) was added to the C-terminus of the IAL protein (IAL^ARL^) by site-directed mutagenesis, no penicillin biosynthetic activity was detected. Since the IAT is only active after an accurate self-processing of the preprotein into α and β subunits, self-processing of the IAL was tested in *Escherichia coli*. Overexpression experiments and SDS-PAGE analysis revealed that IAL is also self-processed in two subunits, but despite the correct processing, the enzyme remained inactive *in vitro*.

**Conclusion:**

No activity related to the penicillin biosynthesis was detected for the IAL. Sequence comparison among the *P. chrysogenum *IAL, the *A. nidulans *IAL homologue and the IAT, revealed that the lack of enzyme activity seems to be due to an alteration of the essential Ser309 in the thioesterase active site. Homologues of the *ial *gene have been found in many other ascomycetes, including non-penicillin producers. Our data suggest that like in *A. nidulans*, the *ial *and *penDE *genes might have been formed from a single ancestral gene that became duplicated during evolution, although a separate evolutive origin for the *ial *and *penDE *genes, is also discussed.

## Background

The β-lactams are one of the most important classes of antibiotics. They are produced by different microorganisms, including filamentous fungi such as *Penicillium chrysogenum *and *Aspergillus nidulans*. These ascomycetes synthesize hydrophobic penicillins using three amino acids as precursors; L-α-aminoadipic acid, L-cysteine and L-valine to form the tripeptide δ (L-α-aminoadipyl)-L-cysteinyl-D-valine (ACV) by the multienzyme ACV synthetase (ACVS), which is encoded by the *pcbAB *gene. This tripeptide is converted into isopenicillin N (IPN), a reaction catalyzed by the IPN synthase (IPNS) encoded by the *pcb*C gene [1]. In the last step of the penicillin pathway, the L-α-aminoadipyl side chain of IPN is substituted by aromatic acyl side chains to form hydrophobic penicillins. This reaction is catalysed by the isopenicillin N acyltransferase (IAT), encoded by the *penDE *gene [2,3]. Previous activation of the aromatic acid by a specific aryl-CoA ligase is required [4,5].

In *P. chrysogenum*, the *pcbAB*, *pcbC *and *penDE *genes are clustered with other ORFs forming an amplifiable DNA unit [6-8]. These other ORFs play only a minor role in the penicillin biosynthesis, since complementation of the npe10 strain (Δ*pen*), which lacks the whole amplified region including the penicillin gene cluster [9,10], with only the *pcbAB*, *pcbC *and *penDE *genes restored full β-lactam synthesis [8,11].

The evolutionary origin of the penicillin gene cluster is intriguing [12]. The first two genes *pcbAB *and *pcbC *do not contain introns despite the large size of *pcbAB *(11 kb); they appear to have been transferred from β-lactam producing bacteria [13-15], unlike the IAT-encoding *penDE *gene, which contains three introns and seems to have been recruited from the fungal genomes.

The last enzyme of the penicillin biosynthetic pathway (IAT) is synthesized as a 40-kDa precursor (proacyltransferase, proIAT), which undergoes an autocatalytic self-processing between residues Gly102-Cys103 in *P. chrysogenum*. The processed protein constitutes an active heterodimer with subunits α (11 kDa, corresponding to the N-terminal fragment) and β (29 kDa, corresponding to the C-terminal region) [16-20]. The IAT has up to five enzyme activities related to penicillin biosynthesis [21]. The substitution of the side chain either occurs directly through the IPN acyltransferase activity, or as a two-step process through the IPN amidohydrolase activity, thus forming 6-aminopenicillanic acid (6-APA) as an intermediate [22].

The *P. chrysogenum *IAT belongs to the N-terminal nucleophile (NTN) family of proteins and it is capable of self-activation (C. García-Estrada and J.F. Martín, unpublished results), as occurs with other NTN amidohydrolases [23]. This enzyme is located inside microbodies (peroxisomes) [24,25] and its transport inside the peroxisomal matrix is not dependent on the processing state of the protein; the unprocessed proIAT variant IAT^C103S ^is correctly targeted to peroxisomes, although it is not active [26].

*In silico *analysis of the *P. chrysogenum *genome revealed the presence of a gene, Pc13g09140, initially described as paralogue of the IAT-encoding *penDE *gene [27]. It was, therefore, of great interest to characterize the *ial *gene at the molecular level and its relationship with the *penDE *gene regarding penicillin biosynthesis.

## Results

### Characterization of the ial gene in *P. chrysogenum*, which encodes a protein (IAL) with high similarity to IAT

**Figure 1:**
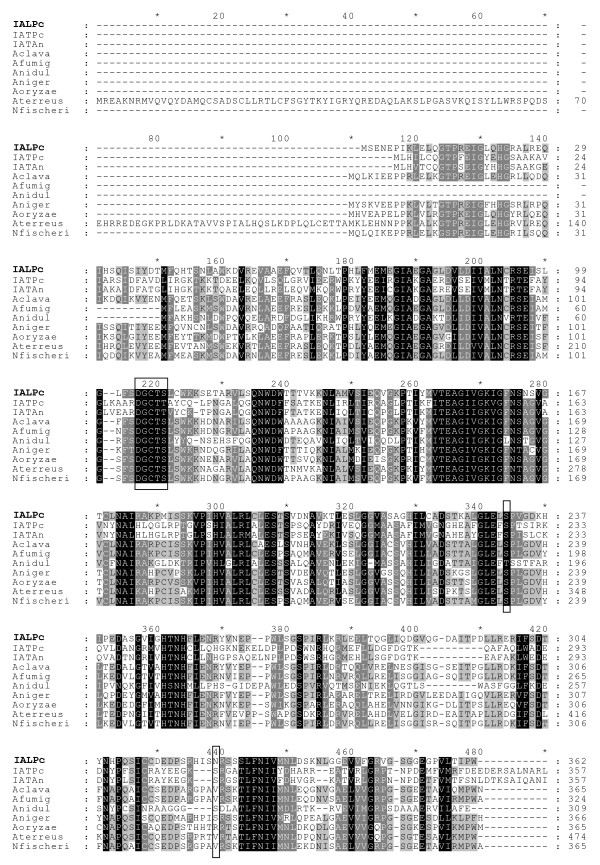
**Alignment of the *P. chryosogenum *IAL (IALPc) to the IATs of *P. chrysogenum *(IATPc) and *A. nidulans* (IATAn) and to different homologues of the IAL present in filamentous fungi such as *A. clavatus *(Aclava), *A. fumigatus *(Afumig), *A. nidulans *(Anidul), *A. niger *(Aniger), *A. oryzae *(Aoryzae), *A. terreus *(Aterreus) and *N. fischeri *(Nfischeri). Those motifs or residues important for IAT enzyme processing or activity are boxed**.

The genome of *P. chrysogenum *Wis54-1255 contains a gene (Pc13g09140) that was initially described as paralogue of the penicillin biosynthetic *penDE *gene [27]. We have confirmed by sequence analysis that this gene is 100% identical to that in the wild-type strain NRRL 1951, indicating that further industrial strain improvement steps have not modified the sequence of this gene. We have termed this gene *ial *because it encodes a protein (IAL for IAT-Like) that shares a 54% similarity (E-value 6e-43, 34% identity) and a 52% similarity (E-value 5e-42, 35% identity) with the IATs of *P. chrysogenum *and *A. nidulans*, respectively. In addition, the IAL showed 81% similarity with an unnamed protein product from *A. oryzae *(GenBank: BAE55742), 80% similarity with a putative IAT of *A. clavatus *(GenBank: XP_001271254), 79% similarity with the hypothetical protein An02g08570 from *A. niger *(GenBank: XP_001399990), 78% similarity with a predicted protein from *A. terreus *(GenBank: XP_001213312), 76% similarity with a putative IAT from *Neosartorya fischeri *(GenBank: XP_001263202), 76% similarity with a putative IAT from *A. fumigatus *(GenBank: XP_754359) and 60% similarity with the hypothetical protein AN6775.2 of *A. nidulans *(GenBank: XP_664379), among others (Fig. 1). The IAL protein is present in several of the sequenced genomes of ascomycetes and deuteromycetes.

It is noteworthy that the *P. chrysogenum *IAL shows some important amino acids and domains that are present in the wild-type IAT, such as the 104 DGCTS 108 motif (equivalent to the 101 DGCTT 105 motif of the IAT containing the G102-C103 processing site) and the S231, which is equivalent to the IAT S227 residue required for IAT cleavage and activity [20]. However, the peroxisomal targeting sequence (PTS1) is absent from the C'-end of the *P. chrysogenum *IAL and related proteins from other filamentous fungi, unlike what is observed in the *P. chrysogenum *and *A. nidulas *IATs, which bear the PTS1 ARL and ANI motifs, respectively (Fig. 1).

### Penicillin biosynthesis is not affected in the *ial *null mutant

**Figure 2:**
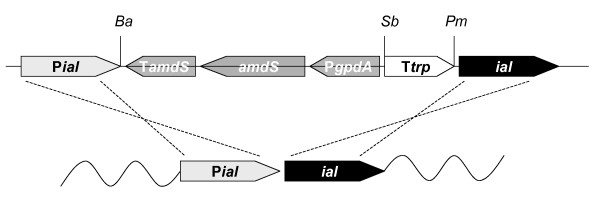
**Generation of the *ial *null mutant in *P. chrysogenum***. The transcription of the *ial *gene was blocked by insertion (double crossover; dashed lines) of the *amdS *selection marker in opposite orientation between the *ial *gene promoter and the *ial *ORF. Restriction enzymes indicated: Ba, BamHI; Sb, SbfI; Pm, PmeI.

In order to test whether the IAL protein participates in the biosynthesis of penicillin in *P. chrysogenum*, we studied the function of the gene in a penicillin high-producing strain, DS17690 [28]. In order to generate null mutants in the *ial *gene without disturbing the genomic context, the *amdS *marker was inserted between the *ial *promoter and its ORF, in the opposite orientation (see Fig. 2). To increase the rate of homologous targeting, a derivative of *P. chrysogenum *DS17690, in which the Non-Homologous End-Joining pathway is disturbed, was used as a host strain. As described for other fungi [29,30] deletion of the *P. chrysogenum *KU70 homologue increases the frequency of homologous recombination significantly (Marco A. van den Berg, unpublished results). Acetamide-consuming transformants were obtained, purified on fresh media and verified for the correct insertion by PCR. Shake flask experiments demonstrated that the *ial *null mutant had no effect on penicillin production in CP medium supplemented with either precursor, adipate or phenylactetate (103 +/- 1% as compared to both DS17690 and DS54465 strains; 100%).

### Expression of the *ial *gene in *P. chrysogenum *and *in vivo *role of the IAL in the benzylpenicillin biosynthetic pathway

**Figure 3:**
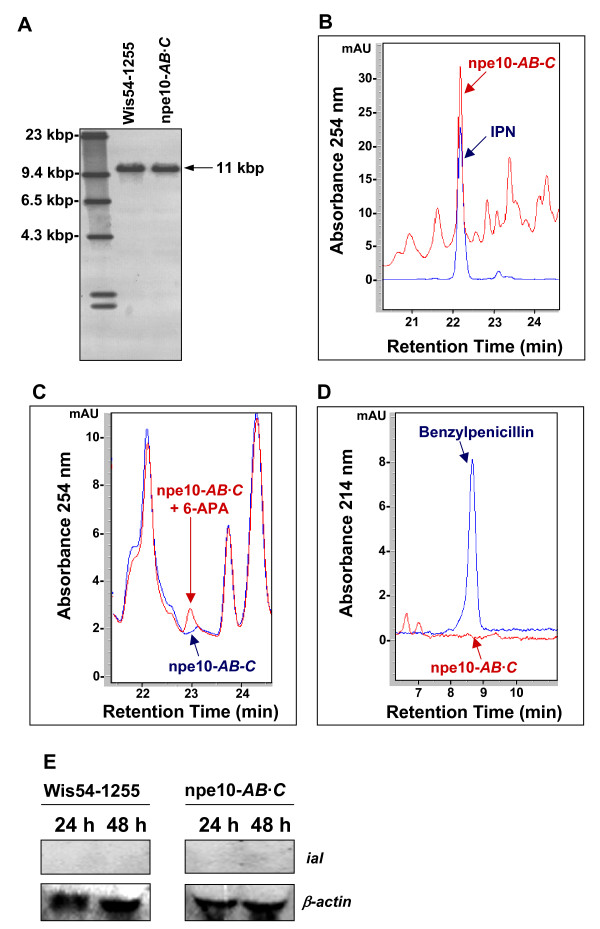
**Characterization and expression of the *ial *gene and *in vivo *activity of the IAL in *P. chrysogenum***. (A) Southern blotting carried out with genomic DNA extracted from the npe-10-*AB*·*C *and Wis54-1255 strains and digested with *Hind*III. The *ial *gene was used as probe. (B) HPLC analysis confirming the production of IPN by the npe10-*AB*·*C *strain. (C) Chromatogram showing the lack of 6-APA production in the npe10-*AB*·*C *strain. (D) Chromatogram showing the lack of benzylpenicillin production in the npe10-*AB*·*C *strain. (E) Northern blot analysis of the *ial *gene expression in npe-10-*AB*·*C *and Wis54-1255 strains. Expression of the *β-actin *gene was used as positive control.

To confirm these results, we carried out different experiments with the engineered strain *P. chrysogenum *npe10-*AB*·*C*. This strain is a transformed derivative of the npe10 PyrG^- ^strain (Δpen) that contains the *pcbAB *and *pcbC *genes, but lacks the wild-type *penDE *gene [11]. Because of these features, this strain is optimal to assess the putative role of the IAL protein in the benzylpenicillin biosynthetic pathway. The integrity of the *ial *gene in the npe10-*AB*·*C *strain was initially tested by PCR (data not shown) and Southern blotting (Fig. 3A). After digestion of the genomic DNA with *Hind*III, one 11-kbp band was observed in the npe10-*AB*·*C*, size that is coincident with that provided by the Wis54-1255 strain digested with the same restriction enzymes (Fig. 3A). However, after sequencing the *ial *gene from the npe10-*AB*·*C *strain, we found a point mutation at nucleotide 980, where C was changed into T (see Discussion). IPN production by the npe10-*AB*·*C *strain was confirmed by HPLC (Fig. 3B). Formation of benzylpenicillin (IPN acyltransferase activity) and 6-APA (IPN amidohydrolase activity) that might be catalyzed by the IAL, were assessed by growing the npe10-*AB*·*C *strain in CP medium. Samples were taken at 48 h and 72 h, but neither 6-APA (Fig. 3C) nor benzylpenicillin (Fig. 3D) were detected by HPLC. This indicates that the npe10-*AB*·*C *strain, which contains the *ial *gene, does not produce these compounds formed in the last step of the penicillin biosynthetic pathway. To test whether the lack of activity is due to a low or null expression rate of the *ial *gene, northern blot experiments were done with samples taken from the npe10-*AB*·*C *and the Wis54-1255 strains grown in CP medium. As shown in Fig. 3E no transcript bands were detected at 24 or 48 h, indicating that this gene is very low or not expressed in *P. chrysogenum*, in agreement with the absence of detectable *ial *mRNA in *P. chrysogenum *NRRL 1951, npe10, Wisconsin54-1255 and DS17690 strains (Marco A. van den Berg, unpublished results).

### Overexpression of the *ial *gene in the *P. chrysogenum *npe10-*AB·C *strain

**Figure 4:**
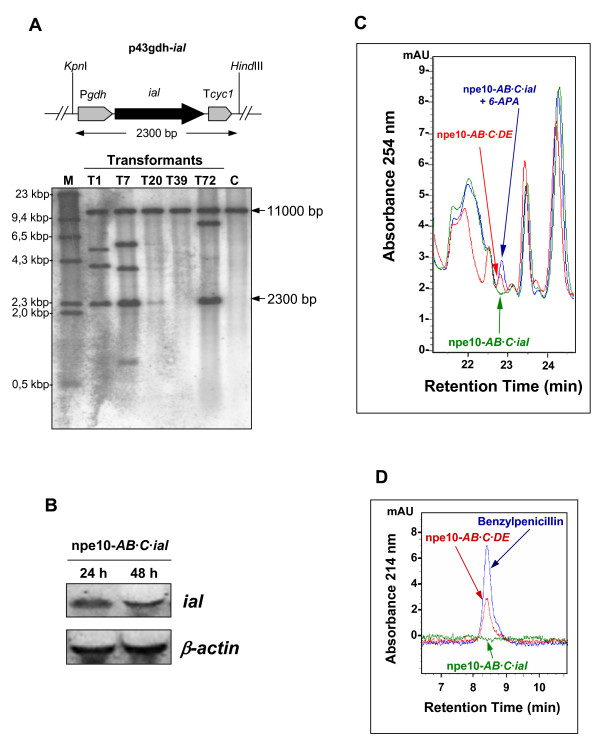
**Overexpression of the *ial *gene in the *P. chrysogenum *npe10-*AB*·*C *strain**. (A) The npe10-*AB*·*C *strain was co-transformed with plasmids p43gdh-*ial *and the helper pJL43b-tTrp. Different transformants were randomly selected (T1, T7, T20, T39 and T72) and tested by Southern blotting after digestion of the genomic DNA with *Hind*III and *Kpn*I. These enzymes release the full P*gdh*-*ial*-T*cyc1 *cassette (2.3 kb) and one 11.0-kb band, which includes the internal wild-type *ial *gene. Bands of different size indicate integration of fragments of the P*gdh*-*ial*-T*cyc1 *cassette in these transformants. Genomic DNA from the npe10-*AB*·*C *strain [C] was used as positive control. The λ-*Hind*III molecular weight marker is indicated as M. (B) Northern blot analysis showing the expression of the *ial *gene in transformant T7 (npe10-*AB*·*C*·*ial *strain). Expression of the *β-actin *gene was used as control. (C) Representative chromatogram of the HPLC analysis of the production of 6-APA by the npe10-*AB*·*C*·*ial *strain. The npe10-*AB*·*C*·*DE *strain was used as positive control. As internal control, 6-APA was added to the samples obtained from the npe10-*AB*·*C*·*ial *strain. (D) Representative chromatogram showing the lack of benzylpenicillin production by the npe10-*AB*·*C*·*ial *strain. Filtrates obtained from the npe10-*AB*·*C*·*DE *strain and a sample of pure potassium benzylpenicillin were used as positive controls.

To assure high levels of the *ial *gene transcript, this gene (without the point mutation at nucleotide 980) was amplified from *P. chrysogenum *Wis54-1255 and overexpressed using the strong *gdh *gene promoter. With this purpose, plasmid p43gdh-*ial *was co-transformed with plasmid pJL43b-tTrp into the *P. chrysogenum *npe10-*AB*·*C *strain. Transformants were selected with phleomycin. Five randomly selected transformants were analyzed by PCR (data not shown) to confirm the presence of additional copies of the *ial *gene in the *P. chrysogenum *npe10-*AB*·*C *genome. Integration of the P*gdh*-*ial*-T*cyc1 *cassette into the transformants of the npe10-*AB*·*C *strain was confirmed by Southern blotting (Fig. 4A) using the complete *ial *gene as probe (see Methods). Transformants T1, T7 and T72 showed the band with the internal wild-type *ial *gene (11 kb) plus a 2.3-kb band, which corresponds to the whole P*gdh*-*ial*-T*cyc1 *cassette. Densitometric analysis of the Southern blotting revealed that 1 copy of the full cassette was integrated in transformant T1, and 3–4 copies in transformants T7 and T72. Additional bands, which are a result of the integration of incomplete fragments of this cassette, were also visible in these transformants. Transformant T7 was randomly selected and expression of the *ial *gene was confirmed by northern blotting using samples obtained from mycelia grown in CP medium (Fig. 4B). This transformant was named *P. chrysogenum *npe10-*AB*·*C*·*ial*.

IPN amidohydrolase (6-APA forming) and IPN acyltransferase (benzylpenicillin forming) activities were tested in this strain under the same conditions used for the northern blot analysis. The npe10-*AB*·*C*·*DE *strain is a derivative of *P. chrysogenum *npe10-*AB*·*C *that expresses the *penDE *gene and has IAT activity [11] and it was used as positive control. Neither 6-APA (Fig. 4C) nor benzylpenicillin (Fig. 4D) were detected in samples taken at 48 h and 72 h from cultures of the transformant T7 grown in CP medium with or without phenylacetic acid, whereas high penicillin production was observed in the control npe10-*AB*·*C*·*DE *strain. This indicates that the IAL protein is not involved in the biosynthesis of penicillin or 6-APA.

### Overexpression of the *ial*^*ARL *^gene containing a modified peroxisomal targeting sequence in the *P. chrysogenum *npe10-*AB*·*C *strain

**Figure 5:**
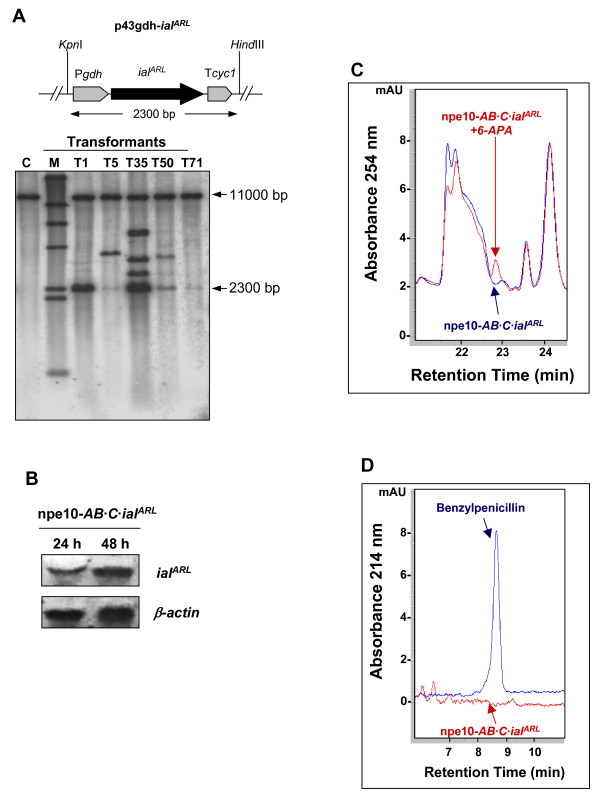
**Overexpression of the *ial*^*ARL *^gene in the *P. chrysogenum *npe10-*AB*·*C *strain**. (A) The npe10-*AB*·*C *strain was co-transformed with plasmids p43gdh-*ial*^*ARL *^and the helper pJL43b-tTrp. Different transformants were randomly selected (T1, T5, T35, T50 and T71) and tested by Southern blotting after digestion of the genomic DNA with *Hind*III and *Kpn*I. These enzymes release the full P*gdh*-*ial*^*ARL*^-T*cyc1 *cassette (2.3 kb) and one 11.0-kb band, which includes the internal wild-type *ial *gene. Bands of different size indicate integration of fragments of the P*gdh*-*ial*^*ARL*^-T*cyc1 *cassette in these transformants. Genomic DNA from the npe10-*AB*·*C *strain [C] was used as positive control. The λ-*Hind*III molecular weight marker is indicated as M. (B) Northern blot analysis showing the expression of the *ial*^*ARL *^gene in transformant T1 (npe10-*AB*·*C*·*ial*^*ARL *^strain). Expression of the *β-actin *gene was used as positive control. (C) Representative chromatogram of the HPLC analysis of the production of 6-APA by the npe10-*AB*·*C*·*ial*^*ARL *^strain. As internal control, 6-APA was added to the samples obtained from the npe10-*AB*·*C*·*ial*^*ARL *^strain. (D) Representative chromatogram showing the lack of benzylpenicillin production by the npe10-*AB*·*C*·*ial*^*ARL *^strain. A sample of pure potassium benzylpenicillin was used as positive control.

One important question is whether the absence of the canonical PTS1 sequence (ARL) at the C-terminal end of the IAL protein and the subsequent mislocalization outside the peroxisomal matrix, is responsible for the lack of activity. Hence, site-directed mutagenesis of the *ial *gene was performed (see Methods) in order to replace the three last amino acids of the IAL protein with the motif ARL. The new construct, p43gdh-*ial*^*ARL *^was co-transformed together with plasmid pJL43b-tTrp into the *P. chrysogenum *npe10-*AB*·*C *strain and transformants were selected with phleomycin. Five randomly selected transformants were analyzed by PCR to confirm the presence of additional copies of the *ial*^*ARL *^gene in the *P. chrysogenum *npe10-*AB*·*C *genome (data not shown). Integration of the P*gdh*-*ial*^*ARL*^-T*cyc1 *cassette into the npe10-*AB*·*C *strain was confirmed in these transformants by Southern blotting (Fig. 5A), using the complete *ial *gene as probe. Transformants T1 and T35 showed the band with the internal wild-type *ial *gene (11 kb) plus a 2.3 kb band, which corresponds to the whole P*gdh*-*ial*^*ARL*^-T*cyc1 *cassette. Additional bands, which are a result of the incomplete integration of this cassette, were also visible in transformant T35. Densitometric analysis of the Southern blotting revealed that 1–2 copies of the full cassette had integrated in transformant T1, and 2–3 copies in transformants T35. Transformant T1 was selected (hereafter named *P. chrysogenum *npe10-*AB*·*C*·*ial*^*ARL*^) and expression of the *ial*^*ARL *^gene was confirmed by northern blotting using samples obtained from mycelia grown in CP medium (Fig. 5B). IPN amidohydrolase and IPN acyltransferase activities were tested under the same conditions used for the northern blot analysis (cultures in CP medium with or without phenylacetic acid). Neither 6-APA (Fig. 5C) nor benzylpenicillin (Fig. 5D) were detected at any time, indicating that the IAL^ARL ^protein is not able to convert IPN into 6-APA or benzylpenicillin even when the PTS1 targeting signal is present.

### Overexpression of the cDNA of the *ial *gene in *E. coli*. The IAL is self-processed, but lacks *in vitro *phenylacetyl-CoA: 6-APA acyltransferase activity

**Figure 6:**
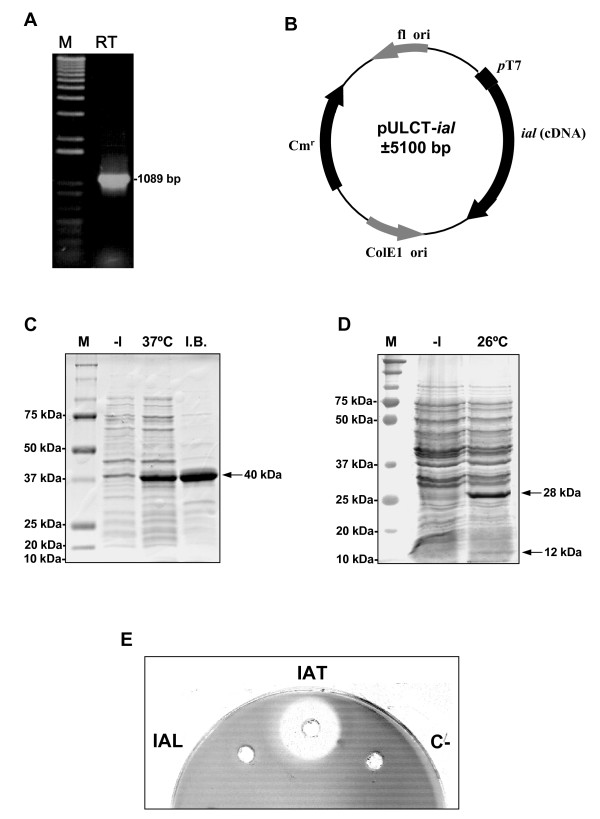
**Characterization of the recombinant IAL in *E. coli***. (A) Agarose gel electrophoresis of the cDNA of the *ial *gene obtained by RT-PCR (RT). The 1-kb Ladder plus molecular marker (Invitrogen) is indicated as M. (B) Schematic representation of plasmid pULCT-*ial*. (C) SDS-PAGE showing the overexpression of the *ial *gene in *E. coli *at 37°C. M: molecular mass marker; -I: uninduced cells; 37°C: total cell extracts obtained after a 5h-induction with IPTG at 37°C; I.B.: inclussion bodies obtained after a 5h-induction with IPTG at 37°C. (D) SDS-PAGE showing the overexpression of the *ial *gene in *E. coli *at 26°C. M: molecular mass marker; -I: uninduced cells; 26°C: soluble cell extracts obtained after a 5h-induction with IPTG at 26°C. Note the lack of the 40-kDa band and the presence of the 28-kDa band. (E) Biossay carried out to determine the *in vitro *phenylacetyl-CoA: 6-APA acyltransferase activity (see Methods) present in the soluble extracts of *E. coli *overexpressing either the *ial *(IAL) or the *penDE *(IAT) genes. As a negative control, the reaction mixture was used without the addition of soluble extracts from *E. coli *overexpressing the *penDE *gene (C-).

In order to analyse the IAL processing and *in vitro *activity, the cDNA of the *ial *gene obtained by RT-PCR as indicated in Methods was overexpressed in *E. coli *JM109 (DE3). One 1089-bp band was amplified (Fig. 6A) and sequenced. Two introns were identified within this gene by comparison of this sequence with the gDNA of the *ial *gene. Intron 1 (61 bp) spanned nucleotides at positions 52–112 of the gDNA, whereas intron 2 (60 bp) spanned positions 518–577 of the gDNA. The cDNA of the *ial *gene was overexpressed using plasmid pULCT-*ial *(see Methods and Fig. 6B). As shown in Fig. 6C, one 40-kDa protein, coincident with the size estimated for the unprocessed IAL protein, was obtained at 37°C. This protein was present in insoluble aggregates forming inclusion bodies. The authenticity of this protein was confirmed by MALDI-TOF peptide mass spectrometry. To test the processing of this protein, the *ial *gene was overexpressed at 26°C, a temperature that is optimal for IAT folding and processing in *E. coli *[26,31]. At this temperature self-processing occurred; no traces of the ~40-Da band were visible and the ~28-kDa and ~12 kDa subunits were obtained in the soluble extracts of *E. coli *(Fig. 6D). We confirmed the processing through the analysis of the ~28-kDa subunit by peptide mass fingerprinting. This peptide was identified as the C-terminal part of the IAL, evidencing that the IAL protein, like the IAT, also undergoes a phenomenon of self-processing.

Once processing was confirmed, *in vitro *activity of the processed IAL protein was assessed (see Methods) using the soluble extracts of *E. coli *obtained after the overexpression of the *ial *gene at 26°C. As positive control, soluble extracts containing the functional processed IAT, obtained from *E. coli *after overepression of the cDNA of the wild-type *penDE *gene at 26°C (using plasmid pPBCαβ as indicated in Methods), were used. Benzylpenicillin formation was tested by bioassay as indicated in Methods. As shown in Fig. 6E, benzylpenicillin was only synthesized in the protein extracts containing the processed wild-type IAT, but not in extract of the processed IAL. This confirms that under *in vitro *conditions, the IAL protein also lacks enzymatic activities related to the biosynthesis of benzylpenicillin, despite the correct self-processing.

## Discussion

The penicillin biosynthetic pathway has been largely elucidated [14,32]. In addition to the three main enzymes involved in this process (ACVS, IPNS and IAT), other ancillary proteins are also required, such as a phenylacetyl-CoA ligases, which primes (activates) the aromatic side chain [4,5] and the phosphopantetheinyl transferase (PPTase), which activates the ACVS and is essential for penicillin biosyntheis in *P. chrysogenum *[33]. The origin of the *pen *gene cluster is intriguing, as occurs with the clusters of other fungal secondary metabolites [12,34]. The first two genes (*pcbAB*, *pcbC*) of the penicillin pathway seem to originate from β-lactam producing bacteria [13], whereas the third gene (*penDE*) encoding the IAT that contains three introns, has been recruited from an ancestral fungal gene [12]. As reported here, *in silico *analysis of the *P. chrysogenum *genome identified a gene (*ial*) paralogue of the *penDE *gene [27] that encodes a protein with high similarity to IAT and is present in most of the genomes of ascomycetes.

We have shown in this work that the *ial *gene is expressed very poorly or not expressed at all in several *P. chrysogenum *strains and that generation of *ial *null mutants does not affect penicillin production. In addition, the *ial *gene in the npe10-*AB*·*C *strain has undergone a point mutation at nucleotide 980 (C to T). After cDNA sequence analysis, this point mutation introduces a stop codon after residue 286, which gives rise to a shorter protein (286 amino acids instead of 362) in the npe10-*AB*·*C *strain. The lack of activity of the IAL present in this strain might be a consequence of the formation of a truncated version derived from the point mutation, but the fact that after overexpression of the *ial *gene (without the point mutation), the IAL protein still lacks both the IPN amidohydrolase and IPN acyltransferase activities *in vivo*, excludes this possibility.

Due to the high homology existing between the IAT and IAL proteins we wondered about the reason for the lack of activity in the IAL. The first possible cause was the absence of the PTS1 peroxisomal targeting motif and the consequent putative mislocalization of the IAL. However, when the PTS1 was added to the C' end of the IAL, this protein was unable to produce 6-APA or benzylpenicillin *in vivo*. Strikingly, it has been recently reported that expression of the *ial *gene homologue in *A. nidulans *(named *aatB*) is easily detected and the protein encoded by this gene contributes to penicillin biosynthesis [35]. The *A. nidulans aatB*-encoded IAL homologue also lacks the canonical PTS1 signal at the C' end, although it is active, indicating that either there might be cryptic PTS1 sequences within this protein as it has been reported in literature [36], or the enzyme is active in the cytosol. The latter possibility is more likely, since addition of the PTS1 signal to the *aatB*-encoded IAL homologue led to an increase in the penicillin titres [35].

The wild-type IAT is only active when it is self-processed into the α (11.5 kDa, pI: 7.24) and β (28.5 kDa, pI: 6.34) subunits [20,26,31]. It is well known that the *P. chrysogenum *and *A. nidulans *IATs differ in their ability to maintain the 40-kDa α-β heterodimer in an undissociated form [31]. Whereas the *P. chrysogenum *proIAT undergoes a quick and efficient self-processing, the *A. nidulans *proIAT remains partially undissociated. This difference in the processing rate of proIAT is responsible, among other reasons, for the low levels of benzylpenicillin production in *A. nidulans *(30-fold less than in wild-type *P. chrysogenum *NRRL1951). We have reported in a previous work that unprocessed proIAT molecules exert a regulatory role generating slow-processing molecules of IAT, thus decreasing the amount of the active form and the penicillin biosynthetic activity [26]. Therefore, the lack of IAL processing might be another explanation for its lack of activity in *P. chrysogenum*. However, when we analysed the sequence of this protein, we found that the G102-C103 processing site of IAT is conserved in the IAL (G105-C106). Self-processing of the IAL was confirmed by MALDI-TOF peptide mass spectrometry after SDS-PAGE analysis of the IAL synthesized in *E. coli *at 26°C. This indicates that the IAL, like the IAT, belongs to the NTN family of proteins, which are capable of self-activation, as it occurs with other NTN amidohydrolases [23,37]. Despite the proper processing, *in vitro *phenylacetyl-CoA: 6-APA acyltransferase activity was not detected, proving that misprocessing is not responsible for the lack of activity.

A detailed analysis of the IAL sequence showed that the amino acid equivalent to the S309 in the IAT, which has been reported to be required for enzyme activity [38], is not conserved in the IAL of *P. chrysogenum *(this amino acid has been replaced by N323). However, in the IAL homologue of *A. nidulans *the amino acid equivalent to the S309 is conserved, indicating that this might be the main reason for the disparity in enzyme activity between the IALs of these two fungi. The S309 is part of the GXS^309^XG motif present in the *P. chrysogenum *and *A. nidulans *IATs and has been previously proposed to be involved in cleavage of phenylacetyl-CoA and binding of the phenylacetyl moiety to form acyl-enzyme molecules [21,31]. The formation of phenylacetyl-enzyme and other acyl-enzyme molecules has been confirmed in the IAT by mass spectrometry [39]. Although the *A. nidulans *IAL does not exactly contain the GXSXG motif, the presence of the Ser272, equivalent to the Ser309, may be sufficient for the activity of this enzyme.

**Figure 7:**
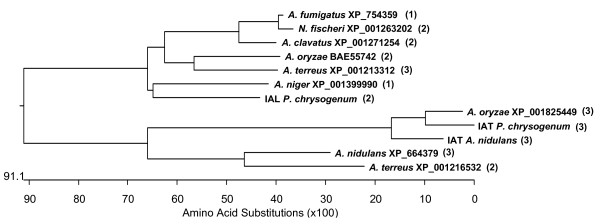
**Phylogenetic tree showing the evolutive distances amongst IATs and putatives IALs from several ascomycetes**. The IAT of *P. chrysogenum *(GenBank: P15802), the IAT of *A. nidulans *(GenBank: P21133) and a hypothetical protein of *A. oryzae *which shares 84% identity with the *P. chrysogenum *IAT (GenBank: XP_001825449), were compared to the *P. chrysogenum *IAL and putative homologues of this protein that are present in different ascomycetes, such as *A. oryzae *(GenBank: BAE55742), *A. clavatus *(GenBank: XP_001271254), *A. niger *(GenBank: XP_001399990), *A. terreus *(GenBank: XP_001213312 and XP_001216532), *N. fischeri *(GenBank: XP_001263202), *A. fumigatus *(GenBank: XP_754359) and *A. nidulans *(*aatB*-encoded protein GenBank: XP_664379). Sequences were aligned using the MegAlign program (Lasergene, DNASTAR, Inc.). Intron content of the genes encoding these proteins is indicated in brackets.

The availability of the genome of several ascomycetes has revealed the presence of *ial *gene homologues in penicillin and non-penicillin producing fungi, whereas the *penDE *gene homologues are only found in penicillin-producing fungi, such as *A. nidulans *and *A. oryzae*. This might indicate that during evolution, a single ancestral gene was duplicated, giving rise to the *penDE *(or *aatA*) gene and its paralogue, the *ial *gene (initially encoding a NTN amidohydrolase not active in *P. chrysogenum *and with low activity in *A. nidulans*). The *P. chrysogenum *IAL and related proteins in other fungi form a separate evolutive clade from IATs (Fig. 7), indicating that they evolved separately. This hypothesis is supported by the presence of duplicated genes encoding putatives IAT and IAL homologues in *A. oryzae*, which also contains the penicillin gene cluster. From those ascomycetes containing this cluster, only *A. nidulans *has an IAL homologue (GenBank: XP_664379) more closely related to IATs, a fact that may explain the presence of penicillin biosynthetic activity in this protein.

Genes encoding IATs in *P. chrysogenum*, *A. nidulans *and *A. oryzae *contain three introns, thus differing from those genes encoding IAL and IAL-homologues (Fig. 7). Only the *aatB *gene encoding the *A. nidulans *IAL homologue and one of the *A. terreus ial *gene homologue (GenBank: XP_001213312), contain three introns. This suggests that alternatively, *ial *and *ial *gene homologues might have had a different origin from the IAT-encoding genes (*penDE *or *aatA *genes), thus encoding proteins with a different function as it was confirmed by the lack of penicillin biosynthetic activity of the *P. chrysogenum *IAL. With this hypothesis, only the *aatB *gene from *A. nidulans *would be a real paralogue of the IAT-encoding gene (*aatA*) formed by gene duplication from a common ancestor. This is supported by the presence of penicillin forming activity of the *aatB*-encoded IAL homologue and by the presence of the same transcription factors binding to the promoter regions of these two genes [35].

## Conclusion

If there was a common ancestor for the *ial *and *penDE *genes, most of the Ascomycota fungi initially had the potential capacity to perform the acyltransferase reaction. However, only a few of them, like *A. nidulans *and *P. chrysogenum*, were able to develop during evolution, the *penDE *encoding the highly functional IAT enzyme. The *penDE *gene was linked to the first two genes (of bacterial origin) of the penicillin pathway, which endowed these microorganisms with an important ecological advantage because of the ability to generate aromatic penicillins. It is likely that the *de novo *formation of this cluster occurred in a common ancestor of the genera *Penicillium *and *Aspergillus*, since the *pen *cluster is present in several species of those genera [40-42]. However, not all genomes of the aspergilli contain the pen cluster; e.g., *A. fumigatus *lacks it, although it contains the *ial *gene. This indicates that the pen cluster might have been horizontally transferred only to some species of the genus, or alternatively, the primitive pen cluster might have been lost during subsequent evolution.

## Methods

### Fungal strains and culture conditions

*P. chrysogenum *NRRL 1951, the natural isolate obtained from an infected cantaloupe [43] was used as wild-type strain. *P. chrysogenum *Wis54-1255, which contains a single copy of the penicillin gene cluster [6], was used as parental strain. *P. chrysogenum *npe10-*AB*·*C *[11], a derivative of the npe10 pyrG^- ^strain (Δpen) [9,10] complemented with the *pcbAB *and *pcbC *genes was used in the molecular analysis of IAT. *P. chrysogenum *DS54465 strain, a derivative of DS17690 [28] wherein the *P. chrysogenum *KU70 homologue has been deleted (Marco A. van den Berg, unpublished results), were used in the *ial *gene deletion experiments.

Fungal spores were collected from plates in Power medium [44] grown for 5 days at 28°C. *P. chrysogenum *liquid cultures were initiated by inoculating fresh spores in complex medium CIM (20 g/l corn steep solids, 10 g/l yeast extract, 58 mM sucrose, 50 mM calcium carbonate, pH 5.7) or defined DP medium [44] without phenylacetate. After incubation at 25°C for 20 h in an orbital shaker (250 rpm), aliquots were inoculated in complex penicillin production CP medium (4 g/l potassium phenylacetate, 20 g/l pharmamedia, 50 g/l lactose, 0.03 M ammonium sulphate, 0.05 M calcium carbonate, pH 6.6) or in defined DP medium with or without phenylacetate (4 g/l).

Spores of the *ial *null mutant were used to inoculate shake flasks with synthetic media supporting β-lactam production [45]. To verify the validity of the findings, two different penicillin side chain precursors were added to the media, phenyl acetic acid and adipate, at 0.3 and 0.5 g/l respectively. Cultivation was for 168 hours at 25°C and 280 rpm. As controls both parent strains, DS17690 and DS54465, were used.

### Plasmid constructs

To completely block the transcription of the *ial *gene, 1500 base pairs of the promoter and the ORF were PCR amplified (for oligonucleotides see the Appendix) and fused to the amdS selection marker, obtained from pHELY-A1 [46] by PCR amplification (Fig. 2). To block eventual read trough from any unconventional transcription start sites in the *amdS *gene, the trp terminator was PCR amplified from plasmid pAMPF21 [47] and inserted between the *amdS *gene and the *ial *ORF (Fig. 2).

Plasmid p43gdh-*ial *was constructed to overexpress the *ial *gene in *P. chrysogenum *starting from plasmid pIBRC43BglII, a derivative of pIBRC43 [48] that contains the *Nco*I restriction site mutated to *Bgl*II. The *ial *gene was amplified from genomic *P. chrysogenum *Wis54-1255 DNA using the primers DElikeF and DElikeR (see the Appendix) and was cloned in the *BglII*-*Stu*I sites of plasmid pIBRC43BglII, between the *A. awamori gdh *gene promoter (a very efficient promoter in ascomycetes) and the *Saccharomyces cerevisiae *cyc1 transcriptional terminator.

Plasmid p43gdh-*ial*^*ARL *^is a derivative of plasmid p43gdh-*ial *and was constructed to overexpress the *ial*^*ARL *^gene in *P. chrysogenum*. This gene includes the sequence encoding the PTS1 (peroxisomal targeting sequece) motif "ARL" at the 3' end, which was introduced using the "QuikChange^® ^Site-Directed Mutagenesis Kit" (Stratagene La Jolla, CA, U.S.A.) following the manufacturer's instructions. Plasmid p43gdh-*ial *was used as template in the PCR reaction performed with HPLC-purified primers ARLF and ARLR (Appendix).

Plasmid pJL43b-tTrp, which contains the *ble *gene (for bleomycin/phleomycin resistance) and the transcriptional terminator of the *A. nidulans trpC *gene, was co-transformed with either p43gdh-*ial *or p43gdh-*ial*^*ARL *^into the Wis54-1255 strain.

Plasmid pPBCαβ has been previously described [26,31] and was used to overexpress the cDNA of the *penDE *gene in *E. coli*.

Plasmid pULCT-*ial *is a derivative of plasmid pULCTαβ [31] and was used to overexpress the *ial *gene in *E. coli*. It was constructed as follows: The cDNA of the *ial *gene was amplified by RT-PCR using primers cDElikeF and DelikeR (Appendix). The RT-PCR product was digested with those endonucleases and subcloned into plasmid pULCTαβ, which was previously digested with *Hind*III, blunt-ended and finally digested with *Nde*I.

### Transformation of *P. chrysogenum *protoplasts

Protoplasts were obtained and transformed as previously described [49,50]. Selection of transformant clones was performed by resistance to phleomycin (30 μg/ml). Selection of acetamide-consuming transformants was done as described previously [51].

### DNA and RNA isolation, Southern and northern blotting

DNA and RNA isolation, Southern and northern blotting were carried out as described before [7]. The *ial *gene was used as probe. The signal provided by the Southern blotting was quantified by densitometry using the "Gel-Pro Analizer" software (Media Cybernetics).

### Intron analysis

Identification of introns in the *ial *gene was performed by RT-PCR using the "OneStep RT-PCR Kit" (Qiagen, Hilden, Germany) following the manufacturer's instructions. Total RNA was extracted from mycelia of the npe10-*AB*·*C*·*ial *strain grown for 48 h in CP medium, using the "RNeasy Mini Kit" columns (Qiagen) following the manufacturer's instructions. RNA was treated with RQ1 RNase-free DNase (Promega Corporation) following the manufacturer's instructions. Oligonucleotides cDElikeF and DElikeR (see the Appendix) were used for this purpose. The presence of introns was confirmed by sequencing.

### Derivatization of IPN and 6-APA and HPLC analysis

Quantification of IPN and 6-APA in *P. chrysogenum *filtrates was carried out by HPLC as previously described [11].

### Extraction and HPLC analyses of penicillin from filtrates

Filtrates or cell extracts (3 ml) were acidified until pH 2.0 with 0.1 N HCl. Benzylpenicillin was extracted by adding n-butyl acetate (3 × 1 ml) and re-extracted from the organic phase with 10 mM phosphate buffer pH 7.5 (3 × 1 ml). This procedure was performed at 4°C. The aqueous phase was lyophilised and resuspended in 300 μl of Milli-Q water for filtrates (in 100 μl for intracellular extracts), which were analysed by HPLC. HPLC analysis of benzylpenicillin was performed in an Agilent 1100 HPLC system with an analytical 4.6 × 150 mm (5 μm) ZORBAX Eclipse XDB-C18 column (Agilent Technologies), a flow rate of 1 ml/min and a detector wavelength of 214 nm. Samples (20 μl) were injected and eluted using as mobile phase Buffer A (30 mM ammonium formate pH 5.0 and 5% acetonitrile) and Buffer B (same as Buffer A plus acetonitrile 20:80, v/v) with an isocratic method (85% of A). Benzylpenicillin showed a retention time of 8.69 ± 0.14 min and its detection limit was 0.1 μg/ml.

### NMR analyses of penicillin from filtrates

Analysis of β-lactams produced by the *ial *null mutant was done by quantitative ^1^H NMR at 600 MHz on a Bruker Avance 600 spectrometer. To a known quantity of filtrate, a known quantity of internal standard (maleic acid), dissolved in phosphate buffer was added prior to lyophilisation. The residue was dissolved in D_2_O and measured at 300 K. The delay between scans (30 s) was more than 5 times T1 of all compounds, so the ratio between the integrals of the compounds of interest and the integral of the internal standard is an exact measure for the quantity of the β-lactams.

### Overexpression of the *penDE *and *ial *genes in *E. coli *and SDS-PAGE of the proteins

The *penDE *and *ial *genes were overexpressed in *E. coli *JM109 (DE3) cells using 0.5 mM IPTG for 6 h at 26°C. Protein samples to be analysed by SDS-PAGE were diluted in loading buffer (60 mM Tris-HCl pH 6.8, 2% SDS, 100 mM DTT, 10% glycerol and 0.1% bromophenol blue), boiled for 5 min, and run in a 12% acrylamide gel. The "Precision Plus Protein All Blue Standards" (Bio-Rad, Hercules, CA, USA), was used as molecular mass marker. Proteins were stained using Coomassie Brilliant Blue R250 dying.

### Determination of the in vitro phenylacetyl-CoA: 6-APA acyltransferase activity

Measurement of the phenylacetyl-CoA: 6-APA acyltransferase activity *in vitro *was carried out using soluble extracts obtained from *E. coli *strains overexpressing either the *penDE *or the *ial *genes. Briefly, 72 μl of cell extracts were mixed with 48 μl of the reaction mixture (0.1 M Tris-HCl pH 8.0, 0.05 M DTT, 0.2 mM 6-APA and 0.2 mM phenylacetyl-CoA) and incubated at 26°C for 15 minutes. The reaction was stopped with 120 μl of methanol, centrifuged at 10,000 × g for 5 minutes and biossayed using *Micrococcus luteus *as test microorganism. Biossays were performed as previously described [26].

## Abbreviations

6-APA: 6-amino penicillanic acid; ACV: δ (L-α-aminoadipyl)-L-cysteinyl-D-valine; ACVS: δ (L-α-aminoadipyl)-cysteinyl-valine synthetase; amdS: acetamidase resistance gene; ble: bleomycin and phleomycin resistance gene; CIM: complex inocullum medium; CP: complex penicillim production medium; DP: defined medium; HPLC: high performance liquid chromatography; IAL: IAT-like; IAT: isopenicillin N acyltransferase; IPN: isopenicillin N; IPNS: IPN synthase; LB: Luria-Bertani broth medium; NTN: N-terminal nucleophile; ORF: open reading frame; Pgdh: glutamate dehydrogenase gene promoter; PPTase: 4'-phosphopantetheinyl transferase; PTS1: peroxisomal targeting sequence type 1; RT-PCR: reverse transcription-PCR; Tcyc1: cytochrome c1 transcriptional terminator; tTrp: tryptophan C biosynthesis gene transcriptional terminator.

## Authors' contributions

CGE and JFM conceived the study and participated in its design. CGE performed the characterization and overexpression experiments. IV made the HPLC analysis of samples. RVU performed the *ial *transcriptional analysis. MAV and RALB carried out the *ial *null mutant experiments. All authors drafted the manuscript and JMF revised the article. All authors read and approved the final manuscript.

## Appendix

Primers used in this work.

**FWD-IAL-LF**; 5'-ccttcgccgactgagtggcatgttgaaccaggacgcctacac-3'

**REV-IAL-LF**; 5'-cggcgctccaacgttgaggataattgctggtcctgtataatgtcagtacaaatacatc-3'

**FWD-IAL-RF**; 5'-atgtctgaaaacgagccaatcaagctggaactc-3'

**REV-IAL-RF**; 5'-ccttcgccgactgatggcttttgagctgaatacttgaagatggagtag-3'

**FWD-amdS**; 5'-ctggaattgtttaaacgcggccgccgcctgcaggataacttcgtatagcatacattatacgaagttatgactctttctggcatgcggagagac-3'

**REV-amdS**; 5'-atggatggatccataacttcgtataatgtatgctatacgaagttatgttgagtggtatggggccatcc-3'

**FWD-TtrpC**; 5'-cgaggagcacctgcaggccgacgccgaccaacaccgcc-3'

**REV-TtrpC**; 5'-ccgccagtgtttaaactagcggccgcatggcgcgccgtattgggtgttacggagcattcac-3'

**DElikeF**; 5'-cattatacagg*agatct*atgtctgaaaac-3'

**DElikeR**; 5'-cagccgtctttca*aggcct*tcaccaggggat-3'

(The *Bgl*II and *Stu*I restriction sequences are italicised)

**ARLF**; 5'-gaagggcctgtgttgaca*gccaggctt*tgaaggcctcggagatcc-3'

**ARLR**; 5'-ggatctccgaggccttca*aagcctggc*tgtcaacacaggcccttc-3'

(The sequence encoding the ARL motif is italicised)

**cDElikeF**; 5'-caggaga*catatg*tctgaaaac-3'

(The *Nde*I restriction site is italicised)
